# Advancing Access to Quality LGBTQIA+ Health Care: Gender Discrimination, Socio-Cultural, and Mental Health Issues: A Mixed-Method Study

**DOI:** 10.3390/ijerph20064767

**Published:** 2023-03-08

**Authors:** Alexandros Argyriadis, Evangelos C. Fradelos, Agathi Argyriadi, Erin Ziegler, Evridiki Kaba

**Affiliations:** 1 School of Health Sciences, Department of Nursing, Frederick University, Nicosia 3080, Cyprus; 2 School of Health Sciences, Department of Nursing, University of Thessaly, 41500 Larisa, Greece; 3 School of Education and Social Sciences, Department of Psychology, Frederick University, Nicosia 1036, Cyprus; 4 Daphne Cockwell School of Nursing, Toronto Metropolitan University, Toronto, ON M5B 2K3, Canada; 5 School of Health Sciences, Department of Nursing, University of West Attica, 11521 Athens, Greece

**Keywords:** LGBTQIA+ community, health equality, healthcare access, cultural competence, soft skills

## Abstract

Recent research highlights the lack of knowledge and reduced skills of health care professionals in communicating with people from the LGBTQIA+ community. This often occurs due to reduced continuing education on social issues in the health sector. The purpose of this research was to study the readiness of health care professionals to manage the social and mental health issues of the LGBTQIA+ community. In particular, the cultural competence of health care professionals targeted at gender identity, the recognition of the level of mastery of soft skills, and the relevant experiences of the participants were studied. For the purposes of conducting this research, a mixed methodology was used to pursue an in-depth study of human beliefs, attitudes, perceptions, ideas, and experiences. More specifically, a previously validated research tool was used to measure cultural competence and assess soft skills. At the same time, interviews were conducted with health care professionals for a more complete understanding of their skills and attitudes. The study comprised a quantitative study involving 479 health care professionals and a qualitative study involving 20 health care professionals, with results from each study. The results showed that the health care professionals’ knowledge of the LGBTQIA+ community is sufficient, but their skills and attitudes towards gender diversity are limited. In addition, the level of acquisition of soft skills by health care professionals is low, and there is insufficient training for health care professionals with regards to social issues. In conclusion, a targeted and structured educational intervention for health care professionals is required to avoid future unfortunate behaviours, and to ensure that the health care provided to healthy and sick populations, regardless of sexual orientation, is adequate.

## 1. Introduction

Current research on health disparities demonstrates that diversity itself is a key predisposing factor for triggering unacceptable behaviours towards minorities. The literature often refers to the fact that established attitudes, stereotypes, and prejudices influence the behaviour of individuals in the dominant group and contribute to the discrimination and social exclusion of different individuals [1]. When the concept of the dominant group is applied to health care professionals, there are significant sociological studies related to the construction of power relations, the maintenance of inequalities, and the dominance of the medical model of thought, among others. Therefore, it is clear that incidents of discrimination, intimidation, and violence against others have historically occupied science to a significant extent [2].

Despite the fact that health care professionals are better educated today, there is social discrimination against the LGBTQIA+ community [3].

In recent years, a significant research trend has been created to study gender discrimination and sexual choices in the health sector, as well as the readiness of health care professionals to adequately manage communication and health care. This discrimination has a negative impact on the LGBTQIA+ community’s health. Specifically, LGBTQIA+ people are at greater risk for cancer, mental illness, and other illnesses and are more likely to smoke, drink alcohol, use drugs, and engage in other risky behaviours [4,5].

Current research states that LGBTQIA+ youth are more likely to experience bullying and report suicidal thoughts and behaviours than non-LGBTQIA+ youth, and bullying is believed to be a precursor to suicide among LGBTQIA+ youth than among non-LGBTQIA+ youth. Another area in which health care professionals are not prepared and trained enough is the process of gender transition and the resulting hormonal imbalance, as well as how to manage such a choice while respecting the client’s rights [6].

The uniqueness and diversity of each individual are inextricably linked to the concept of social stigma, which can lead to social exclusion. Research has shown that several LGBTQIA+ people have been mistreated by their doctor, denied care because of their gender identity, received care from an uncomfortable doctor, or even had to teach their doctor about it [7]. The data emerging from recent research traces the reduced responsiveness of health care professionals to verbal disparagement or belittling in the case of specialists mainly in rural areas who believe they can cure sexual orientation. Scientific research has also shown that LGBTQIA+ people have increased rates of mental health difficulties, substance abuse, and the transmission of HIV and other sexually transmitted infections. The lack of support and health care that LGBTQIA+ people most often face leads to the mismanagement and avoidance of using these services. This further reduces the quality of health of the specific community and potentially increases the cost of care on the state’s side [8].

Moreover, research interest in enhancing the quality of personalised healthcare delivery has recently focused on the cultural competence of health care professionals. Cultural competence is referred to as the process by which the health care provider constantly strives to work effectively according to the patient’s cultural context. This ability includes characteristics such as respect, adapting care to the values, needs, practises, and expectations of individuals, and providing fair and ethical care. These variables affect the health literacy of members of the same ethnic group, health behaviours, perceived risk, attitudes, and beliefs towards health care [9,10].

While there is significant research activity on this specific issue, there seems to be a significant research gap in Greece and Cyprus. Cultural competence has not been linked to the readiness of health care professionals to respond to otherness. There is also a research gap in studies related to the communication between health care professionals and the LGBTQIA+ community. Due to the fact that health is not only a biological process but also includes social and psycho-emotional dimensions, this area of research requires further investigation [9].

## 2. Materials and Methods

The aim of this research was to study the readiness of health care professionals to manage communication issues and offer optimal healthcare to the LGBTQIA+ community. In particular, data was sought regarding the cultural competence and soft skills of health care professionals as a result of the synthesis of literature reports that indicate that these are important components for the inclusion of the “different Other” in the wider social context.

The following research questions emerged after a thorough study of the current literature:

- How culturally competent are health care professionals to manage their communication with people from the LGBTQIA+ community?
- To what extent do health care professionals possess soft skills?
- What is their level of readiness to effectively manage the health needs of LGBTQIA+ patients?

For the purposes of this study, a mixed methodology was used to pursue an in-depth study of human beliefs, attitudes, perceptions, ideas, and experiences. More specifically, the research tool [9] was used to measure cultural competence and assess soft skills, while at the same time, interviews were conducted with health care professionals for a more complete understanding of the skills and attitudes of the participants [9,10]. The design of the semi-structured interview guide was constructed for the needs of the study, which was piloted before its use. The interview guide was structured into three thematic units. In the first thematic unit, the personal data of the participants were recorded, and in the second, emphasis was placed on the cultural competence of health care professionals. In the third thematic unit, the health care professionals’ soft skills were studied. All three sets of questions were designed based on the research questions that emerged from the quantitative study.

### 2.1. Sampling

Our sample included the Greek population since continuous social discrimination and racist behaviours by health care professionals towards people of the LGBTQIA+ community have been shared on social media. The quantitative data collection research tool was already validated, so it was distributed directly online to 479 health care professionals who voluntarily participated in the research process. 16.3% (n = 78) of them were male, and 83.7% (n = 401) were women. None of the participants stated another gender. Of these, 20 were interviewed by the researchers, of which 50% (n = 10) were male and 50% (n = 10) female (Table 1). The snowball sampling was followed so individuals who had already agreed to participate in the study could recommend others to share their experience with the research team. Participants worked in both community and clinical settings and were categorised as a nurse, doctor, or midwife for ease of data collection. Subsequently, and after the collection of the questionnaires, a total of 20 interviews were conducted with participants who agreed to participate in the process. The pilot test resulted in changes and corrections mainly at the verbal level so that the questions were more understandable.

Prior to the interview, a telephone conversation was conducted in which the reasons for the interview were explained, the necessary information was provided, and an email with the consent form was sent. After the oral and written consents were acquired, the interview was conducted and recorded for the purposes of analysis. At the end of the interviews, answers were transcribed and categorised according to the thematic unit to which they belonged. The main findings were evaluated, and a thematic analysis was performed in order to lead the team to the final conclusions.

### 2.2. Data Analysis

Based on the principles of interpretive phenomenology (IP), data analysis was carried out with the goal of illustrating participant experiences and spotting themes and patterns that were expressed in the participants’ own words. The three basic steps of Pietkiewicz and Smith’s [11] data analysis process were carried out in the current study. These steps are (a) detailed, multiple readings of the data and note-making; (b) turning notes into emerging topics; and (c) looking for relationships between topics and grouping. Based on these areas, the data analysis produces a representation of an experience’s meaning based on the identification of significant themes and frequent patterns (themes and patterns), which are captured in the respondents’ own words. There were also notes taken for self-reflection.

### 2.3. Ethics

The importance of the participants’ rights and their voluntary participation in the study was stressed to guarantee that privacy and autonomy were maintained throughout the research process. They were also told that leaving the study at any time would have no effect on them. An emphasis was placed on managing data anonymity and confidentiality prior to, during, and after the interview. The participants were finally informed by the researchers that only the research team could have access to the research data and that it would only be used for research purposes.

### 2.4. Trustworthiness of the Research

Prolonged engagement, persistent observation, triangulation, member checks, thick descriptions, audit trails, researcher’s diaries, and reflexivity are techniques for ensuring the reliability of qualitative studies [11]. The use of reflexivity and triangulation strategies in the current study helped to ensure its reliability. In particular, the researchers’ ability to regulate personal values, perceptions, and prejudices during the study was enabled by the diary they kept throughout the entire time of the qualitative part of the study. The analyst-triangulation method was applied to the triangulation strategy. The data was analysed and interpreted by two researchers, so reliable results could be obtained by cross-referencing the pertinent data. This method helped to reduce the researchers’ potential biases when interpreting the data. After carefully reading the data repeatedly, the study’s emerging themes and subthemes were confirmed.

## 3. Results

The results of this particular study were divided into two categories: (a) the results obtained from the health care professionals’ self-assessment regarding their readiness to manage their collaboration with the LGBTQIA+ community, and (b) the results obtained from the interviews with health care professionals.

The quantitative research involved 479 health care professionals who voluntarily participated in the research process, of whom 16.3% (n = 78) were men and 83.7% (n = 401) were women. None of the participants stated another gender. Of these, 20 were interviewed by the researchers, of which 50% (n = 10) were male and 50% (n = 10) were female.

In the first question from the self-assessment scale that examined whether the participants are aware of possible mistakes they make when communicating with members of the LGBTQIA+ community, 81% (n = 390) of the participants answered that mistakes do not occur, 10.2% (n = 49) that they happen rarely and 8.4% (n = 40) answered often.

In the question of whether they realise that their knowledge of the LGBTQIA+ community is limited and if they would like to learn more, 1.5% (n = 7) answered never, 29.4% (n = 141) sometimes, 60.9% (n = 292) often, and 8.1% (n = 39) said always.

Are they interested in listening well before moving on to the next questions when communicating with a LGBTQIA+ patient? 0.8% (n = 4) answered sometimes, 9.4% (n = 45) often, and 89.8% (n = 430) answered always. No participants chose the “never” option.

I know that differences in sexual orientation are important elements of someone’s identity, and they have equal value. 1.3% (n = 6) answered not at all, 4.2% (n = 20) stated good, 27.1% (n = 130) stated fairly good, and 67.4% (n = 323) answered that they have excellent knowledge.

Do the participants know a lot about the history of the LGBTQIA+ community? 92.3% (442) answered not at all, 7.3% (n = 35) said they have good knowledge, 0.4% (n = 2) answered fairly good, and no one answered excellent.

In the question of whether they recognise that cultures change depending on individuals and time, 1.3% (n = 6) answered sometimes, 4.6% (n = 22) often, and 94.2% (n = 451) answered always.

Asking if the participants are aware that being culturally competent entails continuing education on diversity-related topics 2% (n = 10) answered never, 23% (n = 110) answered sometimes, 15.2% (n = 73) answered often, and 59.7% (n = 286) answered always.

In the question of whether they recognise that stereotypes can encourage exclusion, violence, and injustice 3.5% (n = 17) stated never, 14.8% (n = 71) answered sometimes, 46.3% (n = 222) said often, and 35.2% (n = 169) answered always.

Do the participants know their families’ LGBTQIA+ stories? 63.2% (n = 303) did not know, 35.7% (n = 272) knew some, 1% (n = 5) said they know fairly well, and no one answered that they have excellent knowledge.

The next question was if they deal with potential gaps in their knowledge about LGBTQIA+ and if they try to fill them; 39.7 (n = 190) said never, 44% (n = 211) answered sometimes, 14% (n = 67) answered often, and 2.3% (n = 11) answered always.

I find ways to communicate with people and groups in an appropriate and effective manner, was the next question, in which it was shown that 0.8% (n = 4) answered never, 1.3% (n = 6) answered sometimes, 20.7% (n = 99) answered often, and 77.2% (n = 370) answered always.

In the question of whether the participants intervene effectively when they observe racist behaviour, 44% (n = 211) answered never, 41.3% (n = 198) answered sometimes, 10.2% (n = 49) answered often, and 4.4% (n = 21) answered always.

Can the participants adapt their communication style according to the circumstances and communicate effectively? 8.1% (n = 39) stated that they cannot adapt at all, 12.7% (n = 61) answered somewhat good, 9.4% (n = 45) answered fairly good, and 69.7% (n = 334) answered excellent.

In the question of whether they are looking for opportunities to acquire more transcultural skills, 61.8% (n = 296) said never, 23.2% (n = 111) answered sometimes, 14.6% (n = 70) answered often, and 0.4% (n = 2) answered always.

If they are actively involved in initiatives that promote understanding of different groups, 72.8% (n = 349) answered never, 18.8% (n = 90) answered sometimes, 4.6% (n = 22) stated often, and 3.8% (n = 18) answered always.

Next, when asked if they behave with respect for the culture and opinions of the LGBTQIA+ community, 3.5% (n = 17) chose the “never” option, 4.6% (n = 22) answered sometimes, 50.9 % (n = 244) answered often, and 40.9% (n = 196) answered always.

Do they acquire specialised transcultural knowledge required for their work? 19.8% (n = 95) answered that they do not acquire specialised transcultural knowledge, 25% (n = 120) said they have good knowledge, 14.4% (n = 69) answered fairly good, and 40.7% (n = 195) said they had excellent knowledge.

My colleagues who are characterised as other see me as their ally because I support them, 12.5% (n = 60) said never, 19.4 (n = 93) answered sometimes, 26.9% (n = 129) answered often, and 41% (n = 197) answered always.

I try to understand the needs of others and respect them, even if I disagree with them, 0.2% (n = 1) answered never, 1.7% (n = 7) answered sometimes, 30.6% (n = 124) answered often, and 67.4% (n = 273) answered always.

In the last question, when asked if they like developing friendships, gaining knowledge, and connecting with those who are different from them, 0.7% (n = 3) answered never, 6.9% (n = 28) answered sometimes, 39.3% (n = 159) answered often, and 53.1% (n = 215) answered always.

The questions that revealed statistically significant data were those that asked the participants if they realise that their knowledge about the LGBTQIA+ community was limited and if they would like to learn more (*p* = 0.046), if they were aware that being culturally competent entails continuing education on diversity-related topics (*p* = 0.006), and if they were actively involved in initiatives that promote understanding of different groups (*p* = 0.008). Using correlation analysis, men living in rural places without MSc studies are less culturally competent. Regarding the questions related to the attitudes of health care professionals, the responses of the participants ranged mostly at high levels, meaning that they assessed themselves as having a positive attitude towards LGBTQIA+ people.

**Results from the qualitative part of the study.**

In the qualitative study, 20 healthcare professionals participated. Twelve were women and eight were men; ten were nurses, four were doctors, and there were six psychologists. Seventeen of them had never had any cultural training and twelve of them were living in an urban place compared to eight who lived in a rural area. The analysis of the data revealed three (3) main themes, namely: (a) social discrimination experiences of health care professionals, (b) lack of knowledge of conventional and alternative ways of caring, and (c) soft skills awareness. It is worth noting that the results from the qualitative data present a quite different picture compared to the self-assessment presented earlier. The following are the most indicative results representing each thematic section:

(A) Social discrimination experiences of health care professionals

Participants were initially asked to describe if they had observed any of their colleagues behaving rudely towards a person belonging to the LGBTQIA+ community. It emerged that several incidents of social discrimination occur within clinical settings. The participants’ responses showed an awareness that some of the behaviours of health professionals could be improved, while it is noteworthy that almost all participants described at least one incident of social discrimination.

*There was one time at a hospital where I was working at night and an actor came into the emergency room with abdominal pain. The nurses joked with each other if this pain comes from anal sex. At first I told them that it’s not right to make fun of a person’s sex life. (Nurse 1)*

*Look, I remember several cases where patients come to the hospital to receive help from sexual type accidents. It is something that is of course their choice, but the national health system cannot be burdened with problems that come from choices, not from some disease that cannot be controlled. (Doctor 4)*

(B) Lack of knowledge of conventional and alternative ways of caring

At the same time, when health care professionals were asked about alternative treatment applications for some diseases, such as prostatitis, they appeared to be unaware and at the same time commented negatively. Indicatively, some of the responses that were interviewed about the prostate massage were:

*If there is any such practice and it is even scientifically documented, then I don’t know….*

*Hmm… look, it is not right to see things like this happening. There will definitely be other medicinal methods. (Doctor 2)*

*I must respect the wishes and choices of each patient and make use of all the possible options that exist in the field of Medical science and health. (Nurse 3)*

It was found that most of those who had taboos on various topics in their collaboration and therapeutic communication with people of the LGBTQIA+ community were men, while women appeared to be more open and flexible. Nevertheless, there are cases in which health care professionals show hesitation, fear, ignorance, and prejudice.

(C) Soft skills awareness

The third and last thematic unit on the awareness of soft skills presented data showing either the ignorance of health professionals about what soft skills are or reduced opportunities to develop soft skills. For example, when a participant was asked how important he thought soft skills ere in effectively managing communication with the LGBTQIA+ community, one of the participants replied:

*“This is the first time I’ve received this question. To be honest I don’t know what soft skills are but if this knowledge helps me to be more effective in my work, I am happy to educate myself”. (Psychologist 2)*

To the question about the training and development of soft skills from the place where they work, one participant indicated:

*“Unfortunately, until now the hospital where I work has not presented any training activities to develop soft skills for the staff. I regret to tell you that even in my studies there was little mention on this issue”. (Nurse 1)*

## 4. Discussion

The results obtained from the data analysis showed that health care professionals evaluate themselves positively, that they know about sexual programming and health, have the necessary skills, and can respond excellently in their collaboration with the LGBTQIA+ community. This indicates that health care professionals are well-equipped to provide appropriate care for this population. Furthermore, it suggests that greater efforts should be made to ensure that all members of the LGBTQIA+ community have access to quality health care services [11].

Grouping the answers from the self-assessment tool of cultural readiness for health care professionals into three thematic units (a) knowledge, (b) skills, and (c) attitudes we observe the below mentioned findings.

Participants self-assess their level of knowledge positively and answer most questions, showing that they know about the LGBTQIA+ community. Currently, the literature reports that health care professionals know that the LGBTQIA+ community faces a range of health challenges, including mental health issues, physical health issues, and access to healthcare. Mental health issues such as depression, anxiety, and suicide are common in the LGBTQIA+ community. Physical health issues such as HIV/AIDS, sexually transmitted infections, and cancer are also prevalent in the LGBTQIA+ community. Additionally, recent research states that nurses are well aware of the challenges the LGBTQIA+ community faces with regards to accessing healthcare, such as a lack of insurance, limited access to healthcare services, and few providers who are knowledgeable about LGBTQIA+ health. This lack of access to healthcare can lead to increased health risks and poorer health outcomes for the LGBTQIA+ community. It is essential that healthcare providers strive to create an inclusive environment that is welcoming and respectful of all patients [12].

At the level of skills and attitudes, the participants themselves perceive that they need more support. Similar research shows that health care professionals are positive about change and their continuous professional development. They participate in training seminars, conferences, and other programmes carried out either by the institution in which they work or by external bodies. They understand the importance of adapting to new technologies and processes in order to stay up-to-date with the latest developments in their field. They also recognise the value of engaging with their peers and colleagues to share best practises and insights [13,14].

During the interview process, however, it seems that several participants continue to hold stereotypical beliefs and practise social discrimination towards the LGBTQIA+ community, and their specialised knowledge about the specific health needs of the specific population is lacking. This is concerning, as it indicates that the healthcare system is not fully committed to providing an inclusive environment for all. To ensure that everyone feels welcome and respected, it is essential to provide training on diversity and inclusion topics to all staff members [15]. Indeed, one participant responded that the hospital where he works does not provide training programmes to increase the readiness of the staff. In fact, neither in the previous studies or at the undergraduate or postgraduate level has there been a specialised approach to gender issues [16]. Other published studies agree with this contradiction. At the undergraduate level in various departments of health sciences, intercultural health care is taught, but its focus is on refugees and immigrants. This fact that has been revealed through the study of published detailed study programmes and university curricula [17,18].

## 5. Conclusions

From the literature review, it emerged that in Greece and Cyprus, there are no published articles that study the readiness of health care professionals to effectively manage their collaboration with people from the LGBTQIA+ community. This research focused on three dimensions: How culturally competent are health care professionals to manage their communication with people from the LGBTQIA+ community? To what extent do health care professionals possess soft skills? What is their level of readiness to effectively manage the health needs of LGBTQIA+ patients? Health professionals find it difficult to communicate with diverse communities, and while they seem to have increased knowledge about diversity as well as the desired way of managing diversity, in practise they lack skills and the attitudes adopted are not quite adequate. From the study, it became clear that the soft skills of health professionals are limited. Those who have developed them did so due to their personal initiative and not from educational programmes that are provided either at their undergraduate and postgraduate studies or from the lifelong staff development in their workplace. So, according to the above, health professionals seem unprepared to effectively manage the health needs of LGBTQIA+ patients.

The lack of training can be bridged with special educational programmes and staff development in the future; however, what needs a special approach is the stereotypical thoughts that some health care professionals have about patients with heterogeneity. Some of them use, for example, informal discriminatory practises such as frequent observations or not addressing them at all. The LGBTQIA+ community faces a range of health challenges due to discrimination, stigma, and a lack of access to healthcare. Understanding the historical context of LGBTQIA+ health inequalities, the prevalence of mental and physical health issues, research on the health challenges of the LGBTQIA+ community, healthcare access and delivery, health promotion and education, and nursing care for the LGBTQIA+ community is essential in order to address these health challenges.

## Figures and Tables

**Table 1:** Demographic Characteristics.

Characteristics	N	%		n	%
**Sex**			**Cultural Studies Knowledge**		
Men	78	16.3	yes	23	4.8
Women	401	83.7	no	456	95.2
**Age (years)**			**Place of Residence**		
15–30	79	16.5	urban	223	46.5
31–40	202	42.1	rural	256	53.4
≥40	198	41.3			
**Qualifications**			**Specialty**		
BSc	126	26.3	Nurse	218	45.5
MSc	278	58	Doctor	89	18.6
PhD	1	0.2	Midwife	38	7.9
			Psychologist	38	7.9

Results from the Health Professional Readiness Self-Rating Scale (Table 2).

**Table 2:** Results from the Health Professional Readiness Self-Rating Scale.

Questions	Never/Not at All		Sometimes/ Good		Often/ Fairly Good		Always/ Excellent	
	n	%	n	%	n	%	n	%
I make mistakes in my communication with people who belong to the LGBTQIA+ community	390	81.4	49	10.2	40	8.4	0	0
I realise that my knowledge of the LGBTQIA+ community is limited, and I would like to learn more	7	1.5	141	29.4	292	60.9	39	8.1
I am interested in listening well before moving on to the next questions when communicating with a LGBTQIA+ patient	0	0	4	0.8	45	9.4	430	89.8
I know that differences in sexual orientation are important elements of someone’s identity, and they have equal value	6	1.3	20	4.2	130	27.1	323	67.4
I know a lot about the history of the LGBTQIA+ community	442	92.3	35	7.3	2	0.4	0	0
I recognise that cultures change depending on individuals and time	0	0	6	1.3	22	4.6	451	94.2
I am aware that being culturally competent entails continuing education on diversity-related topics	10	2	110	23	73	15.2	286	59.7
I recognise that stereotypes can encourage exclusion, violence, and injustice	17	3.5	71	14.8	222	46.3	169	35.2
I know family LGBTQIA+ stories	303	63.2	171	35.7	5	1	0	0
I deal with potential gaps in my knowledge of LGBTQIA+ and try to fill them	190	39.7	211	44	67	14	11	2.3
I find ways to communicate with people and groups in an appropriate and effective manner	4	0.8	6	1.3	99	20.7	370	77.2
I intervene effectively when I observe racist behaviour	211	44	198	41.3	49	10.2	21	4.4
I can adapt my communication style according to the circumstances and communicate effectively	39	8.1	61	12.7	45	9.4	334	69.7
I am looking for opportunities to acquire more transcultural skills	296	61.8	111	23.2	70	14.6	2	0.4
I am actively involved in initiatives that promote understanding of different groups	349	72.8	90	18.8	22	4.6	18	3.8
I behave with respect for the culture and opinions of the LGBTQIA+ community	17	3.5	22	4.6	244	50.9	196	40.9
I acquire specialised transcultural knowledge required for my work	95	19.8	120	25	69	14.4	195	40.7
My colleagues who are characterised by diversity see me as their ally because I support them	60	12.5	93	19.4	129	26.9	197	41
I try to understand the needs of others and respect them, even if I disagree with them	1	0.2	7	1.7	124	30.6	273	67.4
I like developing friendships, gaining knowledge, and connecting with those who are different from me	3	0.7	28	6.9	159	39.3	215	53.1

**Results from the qualitative part of the study** **.**

## Data Availability

Not applicable.
